# Association of smoking behaviors with GOLD biological age acceleration and potential mediation: Evidence from NHANES 1999–2023

**DOI:** 10.18332/tid/231597

**Published:** 2026-09-18

**Authors:** Jiaqi Wang, Jiajun He, Yangzhi Li, Zhiqun Yu, Fei Yao, Jimin Zhu, Baikun Li

**Affiliations:** 1 Department of Preventive Medicine, School of Life Sciences, Anhui University of Chinese Medicine, Hefei, China; 2 School of Traditional Chinese Medicine, Anhui University of Chinese Medicine, Hefei, China

**Keywords:** smoking, GOLD BioAge, biological aging, NHANES, mediation analysis

## Abstract

**INTRODUCTION:**

The association between smoking and biological age acceleration, such as KDM and PhenoAge, has been reported, but the dose-response relationship between smoking and the acceleration of GOLD biological age remains unclear. This study aims to explore the association between smoking behavior and the acceleration of GOLD biological age, and to assess the potential mediating roles of oxidative stress and inflammation.

**METHODS:**

This cross-sectional study used data from 12 cycles of NHANES (1999–2023), a survey with a complex multi-stage probability sampling design, and included eligible participants. Smoking exposure (daily amount and status) was assessed via standardized self-report questionnaires. The outcome, GoldBioAge-BAaccel, was calculated as residuals from regressing GOLD biological age on chronological age. Multivariable linear regression and restricted cubic splines were applied to evaluate associations, with subgroup analyses stratified by sex, age, and other covariates, and Wald tests for interaction. Sensitivity analyses were performed to test robustness, and mediation analyses explored the mediating effects of uric acid and C-reactive protein.

**RESULTS:**

The study included 12941 eligible participants. In multivariable linear regression models, each additional cigarette smoked per day was associated with a 0.0763-year increase in BAaccel (β=0.0763; 95% CI: 0.0522–0.1003). Compared with never smokers, current smokers had a 1.5387-year higher BAaccel (β=1.5387; 95% CI: 1.1197–1.9577). Restricted cubic spline analysis showed no evidence of a nonlinear association between CPD and BAaccel (p for nonlinearity=0.285). The association between current smoking and BAaccel differed significantly by age group (p for interaction=0.019). The findings remained consistent across multiple sensitivity analyses. CRP and UA mediated 14.88% and 5.10% of the total effect between daily smoking amount and BAaccel, respectively; for current smoking status (vs never smoking), the corresponding mediated proportions were 9.19 % and 2.40%.

**CONCLUSIONS:**

Smoking behavior is positively associated with GOLD biological age acceleration, and this association is partially mediated by systemic inflammation and oxidative stress.

## INTRODUCTION

Smoking is a major behavioral risk factor for mortality worldwide^1^. Approximately 8 million deaths each year are attributable to tobacco exposure^2,3^, and smokers have a life expectancy at least 10 years shorter than that of non-smokers^4^. Biological age is an integrative measure of physiological status that may reflect health more accurately than chronological age^5,6^ and may improve the assessment of age-related disease and mortality risks^7-9^. In recent years, DNA methylation (DNAm)-based epigenetic clocks, including the GrimAge and PhenoAge clocks, have been widely used to evaluate the association between smoking and biological aging^10-12^. In a study of 2978 middle-aged and older US adults, Klopack et al.^13^ found that lifetime smoking exposure, including adolescent smoking and pack-years, was associated with accelerated GrimAge and PhenoAge. Using NHANES and UK Biobank data, Li et al.^14^ further showed that an unhealthy lifestyle score incorporating smoking, alcohol use, physical inactivity, unhealthy BMI, and poor diet was positively associated with phenotypic age acceleration. Chang and Lin^15^ reported that second-generation epigenetic clocks significantly mediated associations between smoking and diabetes-related outcomes in the Taiwan Biobank and suggested that smoking may impair health directly or indirectly through DNAm changes at aging-related CpG sites.

However, DNAm testing is costly, technically demanding, and difficult to implement in large-scale population screening and primary care, which limits its use in aging assessment and epidemiological surveillance. The recently developed GOLD BioAge algorithm proposed by Hao et al.^16^ may address these limitations. Based on the Gompertz mortality risk model, the algorithm uses LASSO Cox regression to select 9 routine clinical biomarkers, including red blood cell distribution width, albumin, and creatinine, from 26 candidates and combines them linearly to estimate biological age. Unlike Levine’s Phenotypic Age (PhenoAge), which relies on an inverse cumulative distribution function and a double-logarithmic transformation, GOLD BioAge uses a more direct linear formulation based on the Gompertz risk function. In benchmark analyses of NHANES and UK Biobank data, GOLD BioAge showed slightly better discrimination for all-cause mortality than existing aging measures. In NHANES, its C-index was 0.829, compared with 0.827 for PhenoAge and 0.807 for the Klemera–Doubal method (KDM) biological age. Corresponding values in the UK Biobank were 0.784, 0.776, and 0.775, respectively. GOLD BioAge may therefore offer advantages in predictive performance, computational simplicity, and clinical interpretability. The original study^16^ also showed that GOLD BioAge captured accelerated aging associated with unhealthy lifestyles, including smoking, suggesting its potential value as an outcome measure for smoking-related biological aging. Nevertheless, no study has specifically quantified associations between smoking behaviors and GOLD BioAge acceleration.

Therefore, using nationally representative NHANES data from 1999 to 2023, this study systematically assessed the association between smoking behavior (daily smoking amount and smoking status) and GOLD BioAge acceleration, and further examined the mediating effects of chronic inflammation and oxidative stress through mediation analysis, aiming to offer novel epidemiological insights into how smoking accelerates biological aging.

## METHODS

### Data source and study population

This study constitutes a secondary analysis of cross-sectional data drawn from the National Health and Nutrition Examination Survey (NHANES), comprising 12 consecutive cycles (1999–2023) and administered by the National Center for Health Statistics (NCHS) of the Centers for Disease Control and Prevention. NHANES employs a complex, multistage probability sampling design to obtain a nationally representative sample of the non-institutionalized US civilian population. NHANES data are publicly available from the CDC website (https://wwwn.cdc.gov/nchs/nhanes/).

The survey protocols were approved by the NCHS Research Ethics Review Board, and all participants provided written informed consent. The initial sample size of this study was 103284 individuals. The inclusion criteria were: age ≥18 years, non-pregnancy, valid MEC weight (WTMEC2YR >0), complete smoking questionnaire data, no missing values for the 9 biochemical indicators required for calculating GOLD BioAge, and BAaccel values within the normal physiological range (-15 to 15 years). A total of 12941 individuals were finally included in the analysis to explore the association between smoking status and accelerated biological age. Because CPD (cigarettes per day) data were unavailable for 3180 former smokers, these participants were excluded from analyses of CPD.

### Assessment of GOLD BioAge acceleration

GOLD BioAge is calculated as a linear combination of 9 routinely measured clinical biomarkers: albumin, creatinine, fasting blood glucose, lymphocyte percentage, mean corpuscular volume, red blood cell distribution width, alkaline phosphatase, white blood cell count, and γ-glutamyl transferase^16^. Biological age acceleration (BAaccel) was defined as the residual from a linear regression of GOLD BioAge on chronological age. A BAaccel value >0 indicates that an individual’s biological age is higher than expected for their chronological age, whereas a value <0 indicates a biological age lower than expected. BAaccel is expressed in years.

### Assessment of smoking exposure

Smoking exposure was assessed using 2 measures. First, CPD was obtained from questionnaire item SMD650, which records the average number of cigarettes smoked per day on smoking days during the past 30 days. Participants were categorized as non-smokers, light smokers (1–10 cigarettes/day), moderate smokers (11–20 cigarettes/day), or heavy smokers (>20 cigarettes/day) according to clinical criteria^17^. Second, smoking status was determined from items SMQ020 (smoked ≥100 cigarettes during one’s lifetime) and SMQ040 (current smoking status) and categorized as never smoking (<100 cigarettes during one’s lifetime), former smoking (≥100 lifetime cigarettes but not currently smoking), or current smoking (≥100 lifetime cigarettes and currently smoking)^18^. Serum cotinine (LBXCOT) was used as an objective biomarker to assess the reliability of self-reported smoking information. A cotinine concentration >15 ng/mL was considered indicative of active smoking^19^. Serum cotinine levels exhibited a marked rightskewed distribution; therefore, a natural logarithmic transformation was applied in the continuous sensitivity analysis to correct for skewness.

### Potential covariates

Potential covariates were selected on the basis of established correlates of smoking and potential confounders^18,20,21^. Demographic characteristics included sex (male or female), age (years), and race/ethnicity (Mexican American, other Hispanic, non-Hispanic White, non-Hispanic Black, or other races). Socioeconomic status was assessed using education level (lower than high school, high school or equivalent, or college or higher) and the family poverty-to-income ratio (PIR). Lifestyle factors included body mass index (BMI, kg/m^2^), alcohol consumption status (drinker or non-drinker), and physical activity level, expressed as metabolic equivalents derived from the Physical Activity Questionnaire (PAQ-MET). Clinical covariates included hypertension, diabetes, and dyslipidemia, each categorized as yes or no. Marital status (married, unmarried, or widowed/divorced/separated)^22^ and NHANES survey cycle (cycles A–L, 1999–2023) were also included to account for systematic variation across survey periods. Chronological age was not included as a covariate because BAaccel was defined as the age-residualized value of GOLD BioAge.

### Statistical analysis

This study’s analysis is based on the complex multistage probability sampling design of NHANES, and sampling weights are included to obtain nationally representative estimates. The weights used in the analysis are the MEC examination weights (WTMEC2YR) divided by the combined number of cycles (i.e. 12), to reflect the sampling structure of the multi-cycle combined data from 1999 to 2023. Baseline characteristics are described based on weighted data, continuous variables are expressed as weighted means (95% CI), and comparisons between groups are conducted using the Kruskal-Wallis rank sum test based on the complex sampling design; categorical variables are expressed as weighted percentages (95% CI), and comparisons between groups are conducted using the Rao-Scott corrected χ^2^ test.

Ordinary least squares regression with cluster-robust standard errors was the primary method used to evaluate associations between smoking behaviors and BAaccel. Confidence intervals were based on the t-distribution, and survey-weighted generalized linear models (GLMs) were used to assess the sensitivity of variance estimates. The multicollinearity among the covariates included in the fully adjusted model was evaluated using the variance inflation factor (VIF) and its generalized form (GVIF). For continuous variables and binary variables, VIF was used, with the criterion of VIF <5; for multi-category variables, the standardized form of the generalized variance inflation factor, GVIF^1/2df^ , was adopted with a threshold of <2 as the criterion for no severe multicollinearity. Restricted cubic splines, specified as natural splines with 4 degrees of freedom (df), were fitted within survey-weighted GLMs, and regTermTest was used to assess nonlinearity. Subgroup analyses and Wald tests for interaction were performed by sex, age, education level, income, alcohol use, and BMI. Missing covariate data were handled using multiple imputation by chained equations (MICE) under the missing at random assumption. The proportions of missing values were 17.42% for marital status, 9.65% for poverty income ratio, 9.72% for physical activity, 2.36% for education level, and 1.12% for body mass index. The primary analysis was based on m=20 imputed datasets, with a sensitivity analysis using m=5 to assess the robustness of the estimates. Exposure and outcome variables were not imputed, and estimates were pooled using Rubin’s rules. A stepwise adjustment strategy was used to construct three successive models. Model 1 was a crude model that included only the exposure variables without covariate adjustment. Model 2 additionally adjusted for sex, race, education level, poverty income ratio, body mass index, and marital status. Model 3 (the fully adjusted model) further included survey cycle, alcohol consumption, diabetes, hypertension, hyperlipidemia, and physical activity level. In addition, a series of sensitivity analyses were performed, including alternative definitions of exposure, exclusion of extreme populations and patients with specific diseases, Evalue analysis for unmeasured confounding, and use of an alternative outcome (BioAge–Age). All these analyses were conducted in parallel for both CPD and smoking status as the exposure measures. The mediation analysis was conducted using the R mediation package (with 1000 bootstrap iterations) to evaluate the mediating effects of uric acid and C-reactive protein in the association between CPD and smoking status (current vs never) and BAaccel. This package currently does not support complex sampling weights, and the mediation analysis did not include survey weight adjustment. All analyses were conducted using R version 4.5.2. All tests were 2-sided, and p<0.05 was considered statistically significant.

## RESULTS

### Sample selection process for the research

**Figure 1:**
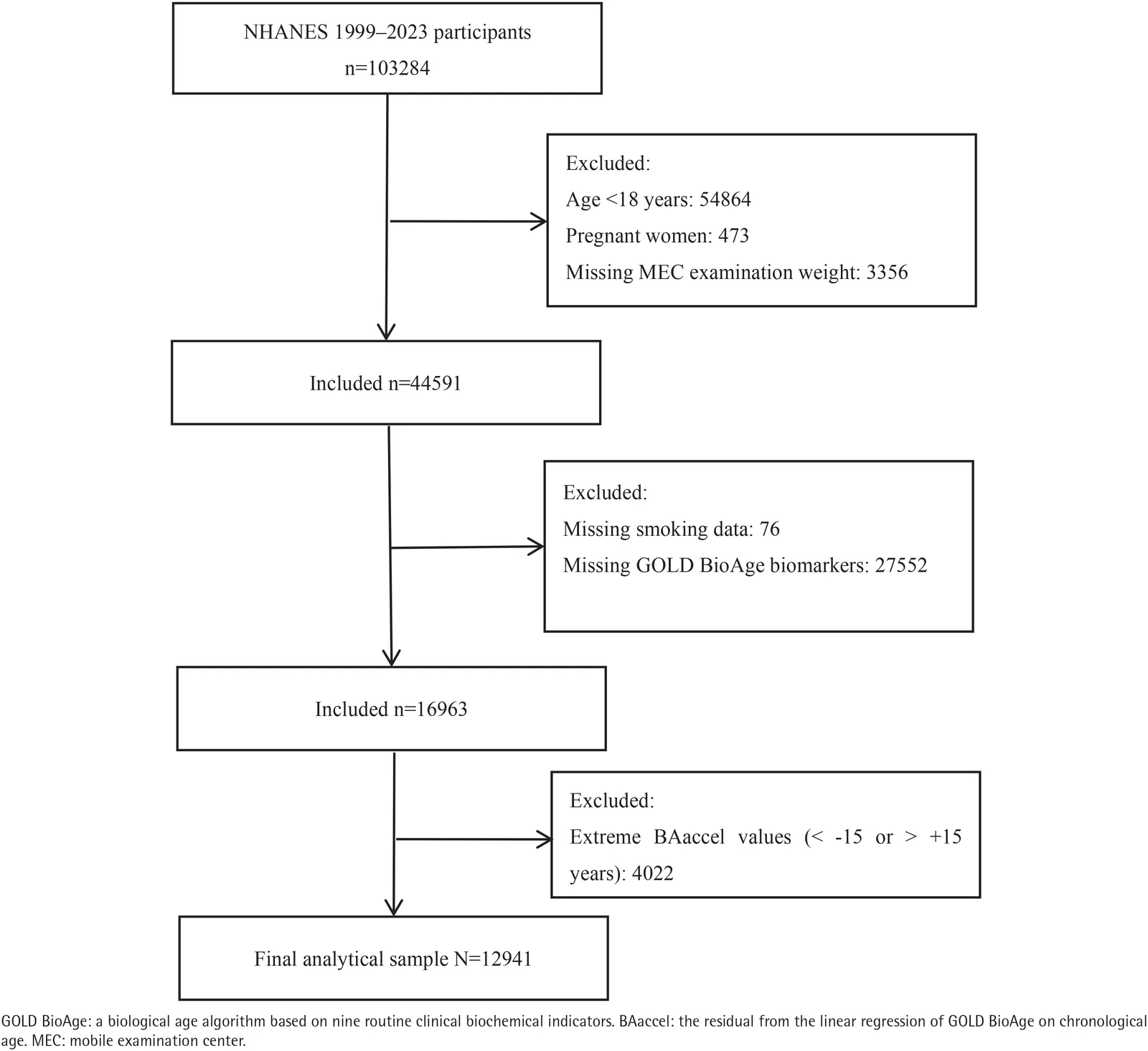
Flow chart of study population selection, NHANES 1999–2023 (N=12941)

From the initial 103284 participants, exclusions were made for those aged <18 years (n=54864), pregnant women (n=473), participants without valid MEC weights (WTMEC2YR=0, n=3356), those with missing smoking data (n=76), those missing any of the nine GOLD BioAge biomarkers (n=27552), and extreme BAaccel outliers (< -15 or > +15 years, n=4022), resulting in a final sample of 12941 individuals for analysis (Figure 1).

### Characteristics of the study population

The CPD analysis included 9761 participants, who were classified into 4 groups according to smoking intensity. The groups differed significantly in age, PIR, BMI, serum cotinine concentration, sex, race/ethnicity, education level, marital status, alcohol use, and diabetes prevalence (all p<0.05). The mean ages of the non-smoking, 1–10, 11–20, and >20 cigarettes/day groups were 46.51 (95% CI: 45.86–47.16), 42.53 (95% CI: 41.41–43.66), 45.92 (95% CI: 44.87–46.97), and 48.57 (95% CI: 46.87–50.27) years, respectively, with significant between-group differences (p<0.001). With increasing daily cigarette consumption, the proportions of males and drinkers, as well as serum cotinine levels, increased progressively (all p<0.001), whereas the proportion of individuals with a college degree or higher decreased (p<0.001). Physical activity, BAaccel, hypertension prevalence, and dyslipidemia prevalence did not differ significantly among the groups (all p>0.05) (Table 1).

**Table 1:** Survey-weighted baseline characteristics of study participants, by daily cigarette consumption, NHANES 1999–2023 (N=9761)

*Variables*	*Non-smokers*	*1–10*	*11–20*	*>20*	*p*
Sample size, n	7365	1419	760	217	
	***Weighted Mean (95% CI)***				
Age (years)	46.51 (45.86–47.16)	42.53 (41.41–43.66)	45.92 (44.87–46.97)	48.57 (46.87–50.27)	<0.001
Poverty income ratio	3.15 (3.08–3.23)	2.35 (2.22–2.48)	2.53 (2.34–2.71)	2.53 (2.12–2.93)	<0.001
Body mass index (kg/ m²)	28.80 (28.53–29.07)	27.95 (27.47–28.44)	27.24 (26.71–27.77)	27.62 (26.36–28.89)	<0.001
Physical activity (METmin/week)	386.37 (374.30–398.44)	389.15 (347.06–431.24)	410.15 (344.77–475.54)	376.75 (340.28–413.22)	0.1614
Serum cotinine (ng/mL)	10.38 (7.44–13.32)	179.05 (165.17–192.92)	280.76 (262.64–298.88)	303.86 (272.82–334.89)	<0.001
Biological age acceleration (years)	-0.97 (-1.26 – -0.67)	-0.36 (-0.92–0.19)	-0.49 (-1.36–0.38)	-0.49 (-1.71–0.74)	0.1558
	***Weighted % (95% CI)***				
**Sex**					<0.001
Male	42.5 (41.2–43.9)	54.0 (50.8–57.1)	53.9 (49.4–58.4)	77.2 (70.5–82.7)	
Female	57.5 (56.1–58.8)	46.0 (42.9–49.2)	46.1 (41.6–50.6)	22.8 (17.3–29.5)	
**Race/ethnicity**					<0.001
Mexican American	10.2 (8.7–12.0)	9.7 (7.9–11.9)	1.9 (1.1–3.3)	1.3 (0.4–3.6)	
Other Hispanic	7.3 (6.3–8.6)	7.2 (5.7–9.1)	3.1 (2.0–4.7)	1.8 (0.8–4.1)	
Non-Hispanic White	62.2 (59.6–64.8)	56.7 (52.6–60.8)	81.0 (76.8–84.5)	88.6 (84.0–92.0)	
Non-Hispanic Black	10.7 (9.4–12.1)	17.2 (14.7–20.1)	7.2 (5.6–9.2)	3.5 (1.9–6.2)	
Other race/ethnicity	9.5 (8.4–10.9)	9.2 (7.0–11.9)	6.9 (4.7–10.0)	4.9 (2.7–8.5)	
**Education level**					<0.001
Lower than high school	13.0 (11.8–14.3)	24.5 (21.2–28.1)	21.8 (18.6–25.4)	31.4 (22.6–41.8)	
High school or equivalent	20.2 (18.8–21.7)	29.1 (25.9–32.5)	35.2 (30.8–39.8)	33.9 (25.4–43.5)	
College or higher	66.8 (64.9–68.7)	46.4 (42.4–50.4)	43.0 (38.6–47.5)	34.8 (26.5–44.1)	
**Marital status**					<0.001
Married/living with partner	65.2 (63.3–67.0)	53.4 (49.8–57.1)	57.7 (52.4–62.7)	60.5 (51.3–69.0)	
Never married	17.2 (15.9–18.5)	25.0 (22.1–28.2)	17.9 (14.4–22.0)	14.4 (9.6–21.2)	
Widowed/divorced/separated	17.6 (16.3–19.1)	21.5 (18.9–24.5)	24.4 (20.7–28.6)	25.0 (18.8–32.5)	
**Alcohol consumption**					<0.001
Yes	41.3 (38.9–43.8)	64.0 (60.3–67.6)	65.1 (60.4–69.5)	69.4 (59.9–77.5)	
No	21.4 (19.5–23.4)	7.1 (5.7–8.8)	8.7 (6.5–11.5)	13.1 (7.9–21.2)	
Unknown	37.3 (34.5–40.2)	28.9 (25.4–32.7)	26.3 (22.2–30.9)	17.5 (11.2–26.2)	
**Diabetes**					0.0263
No	91.4 (90.4–92.2)	93.4 (91.6–94.9)	94.2 (91.7–95.9)	89.5 (83.1–93.7)	
Yes	8.6 (7.8–9.6)	6.6 (5.1–8.4)	5.8 (4.1–8.3)	10.5 (6.3–16.9)	
**Hypertension**					0.1427
No	71.5 (70.1–72.9)	70.8 (67.6–73.8)	69.5 (64.8–73.7)	63.3 (55.5–70.5)	
Yes	28.5 (27.1–29.9)	29.2 (26.2–32.4)	30.5 (26.3–35.2)	36.7 (29.5–44.5)	
**Hyperlipidemia**					0.4651
No	54.7 (52.5–56.9)	56.4 (52.7–60.1)	57.1 (52.5–61.5)	50.7 (42.9–58.5)	
Yes	45.3 (43.1–47.5)	43.6 (39.9–47.3)	42.9 (38.5–47.5)	49.3 (41.5–57.1)	

Sample sizes (n) are unweighted counts. Continuous variables are presented as survey-weighted means (95% CIs), with between-group comparisons performed using the Kruskal–Wallis rank-sum test accounting for the complex survey design. Categorical variables are presented as survey-weighted percentages (95% CIs), with between-group comparisons performed using Rao–Scott corrected chi-squared tests. The 95% CIs were calculated using the t distribution with survey design correction. MEC examination weights divided by the number of survey cycles (WTMEC2YR/12) were used as sampling weights. Biological age acceleration was defined as the residual from a linear regression of GOLD BioAge on chronological age. MET: metabolic equivalent of task. CI: confidence interval. A two-sided p<0.05 was considered statistically significant.

The smoking-status analysis included 12941 participants, who were classified as never, former, or current smokers. The 3 groups differed significantly in age, PIR, BMI, physical activity, serum cotinine concentration, BAaccel, sex, race/ethnicity, education level, marital status, and alcohol use (all p<0.001). The former smoking group exhibited the lowest biological age acceleration (-1.64 years, 95% CI: -2.02 – -1.25), followed by the non-smoking group (-0.97 years, 95% CI: -1.26 – -0.67) and the current smoking group (-0.46 years; 95% CI: -0.88 – -0.03), with a significant overall difference (p<0.001). Notably, the former smoking group was the oldest on average (54.56 years, p<0.001) and had the highest rates of hypertension (40.2%) and diabetes (12.3%) (both p<0.001). Daily smoking amount was positively associated with serum cotinine levels (p<0.001) and inversely associated with college education (p<0.001) (Table 2).

**Table 2:** Survey-weighted baseline characteristics of study participants, by smoking status, NHANES 1999–2023 (N=12941)

*Variables*	*Non-smokers*	*Former smokers*	*Current smokers*	*p*
Sample size, n	7365	3161	2415	
	***Weighted Mean (95% CI)***			
Age (years)	46.51 (45.86–47.16)	54.56 (53.69–55.43)	44.46 (43.72–45.20)	<0.001
Poverty income ratio	3.15 (3.08–3.23)	3.24 (3.14–3.34)	2.43 (2.31–2.56)	<0.001
Body mass index (kg/m²)	28.80 (28.53–29.07)	29.63 (29.30–29.95)	27.65 (27.32–27.97)	<0.001
Physical activity (MET-min/week)	386.37 (374.30–398.44)	404.68 (384.37–424.99)	394.63 (362.27–426.99)	<0.001
Serum cotinine (ng/mL)	10.38 (7.44–13.32)	34.53 (24.14–44.91)	229.47 (216.07–242.86)	<0.001
Biological age acceleration (years)	-0.97 (-1.26 – -0.67)	-1.64 (-2.02 – -1.25)	-0.46 (-0.88 – -0.03)	<0.001
	***Weighted % (95% CI)***			
**Sex**				<0.001
Male	42.5 (41.2–43.9)	58.3 (55.8–60.8)	56.6 (54.2–58.9)	
Female	57.5 (56.1–58.8)	41.7 (39.2–44.2)	43.4 (41.1–45.8)	
**Race/ethnicity**				<0.001
Mexican American	10.2 (8.7–12.0)	6.6 (5.5–7.9)	6.0 (4.9–7.4)	
Other Hispanic	7.3 (6.3–8.6)	4.8 (3.8–6.0)	5.1 (4.0–6.4)	
Non-Hispanic White	62.2 (59.6–64.8)	77.4 (74.8–79.8)	69.0 (65.8–72.1)	
Non-Hispanic Black	10.7 (9.4–12.1)	5.9 (5.1–6.8)	12.0 (10.3–13.9)	
Other race/ethnicity	9.5 (8.4–10.9)	5.4 (4.4–6.5)	7.8 (6.3–9.7)	
**Education level**				<0.001
Lower than high school	13.0 (11.8–14.3)	14.4 (12.7–16.1)	24.3 (21.6–27.2)	
High school or equivalent	20.2 (18.8–21.7)	22.4 (20.2–24.8)	31.8 (29.2–34.5)	
College or higher	66.8 (64.9–68.7)	63.2 (60.3–66.0)	43.9 (40.7–47.2)	
**Marital status**				<0.001
Married/living with partner	65.2 (63.3–67.0)	71.2 (68.8–73.5)	56.0 (53.0–58.9)	
Never married	17.2 (15.9–18.5)	9.0 (7.6–10.7)	21.1 (19.0–23.5)	
Widowed/divorced/separated	17.6 (16.3–19.1)	19.8 (17.7–22.0)	22.9 (20.5–25.5)	
**Alcohol consumption**				<0.001
Yes	41.3 (38.9–43.8)	58.7 (55.8–61.5)	64.8 (62.3–67.3)	
No	21.4 (19.5–23.4)	9.2 (8.0–10.5)	8.3 (6.9–10.0)	
Unknown	37.3 (34.5–40.2)	32.1 (29.4–35.0)	26.9 (24.2–29.7)	
**Diabetes**				<0.001
No	91.4 (90.4–92.2)	87.7 (86.1–89.0)	93.3 (91.8–94.5)	
Yes	8.6 (7.8–9.6)	12.3 (11.0–13.9)	6.7 (5.5–8.2)	
**Hypertension**				<0.001
No	71.5 (70.1–72.9)	59.8 (57.4–62.2)	69.4 (67.0–71.8)	
Yes	28.5 (27.1–29.9)	40.2 (37.8–42.6)	30.6 (28.2–33.0)	
**Hyperlipidemia**				<0.001
No	54.7 (52.5–56.9)	48.6 (45.6–51.7)	56.2 (53.5–58.8)	
Yes	45.3 (43.1–47.5)	51.4 (48.3–54.4)	43.8 (41.2–46.5)	

Sample sizes (n) are unweighted counts. Continuous variables are presented as survey-weighted means (95% CIs), with between-group comparisons performed using the Kruskal–Wallis rank-sum test accounting for the complex survey design. Categorical variables are presented as survey-weighted percentages (95% CIs), with between-group comparisons performed using Rao–Scott corrected chi-square tests. The 95% CIs were calculated using the t-distribution with survey design correction. MEC examination weights divided by the number of survey cycles (WTMEC2YR/12) were used as sampling weights. Biological age acceleration was defined as the residual from a linear regression of GOLD BioAge on chronological age. MET: metabolic equivalent of task. CI: confidence interval. A two-sided p<0.05 was considered statistically significant.

### Associations between smoking behaviors and BAaccel

In Model 1, daily smoking amount was not significantly associated with BAaccel (β=0.0185; 95% CI: -0.0024–0.0394, p=0.082). Compared with never smokers, former smokers had a significantly lower BAaccel by -0.7554 years (β= -0.7554, 95% CI: -1.0504 – -0.4604, p<0.001), whereas current smokers showed no significant difference (β=0.4228; 95% CI: -0.0637–0.9094, p=0.089). In Model 2, each additional cigarette per day was associated with a 0.069-year increase in BAaccel (β=0.069; 95% CI: 0.0466–0.0915, p<0.001). Relative to never smokers, former smokers did not differ significantly (β= -0.1043; 95% CI: -0.3430–0.1345, p=0.392), while current smokers had a 1.3396-year higher BAaccel (β=1.3396; 95% CI: 0.9284–1.7508, p<0.001). In the fully adjusted model (Model 3), each additional cigarette smoked per day was associated with a 0.0763year increase in BAaccel (β=0.0763, 95% CI: 0.0522–0.1003, p<0.001). This association remained robust after multiple imputation by chained equations (MICE) for missing data (β=0.076; 95% CI: 0.0486–0.1033, p<0.001). Compared with never smokers, former smokers showed no significant difference in BAaccel (β= -0.0546, 95% CI: -0.3381–0.2288, p=0.706), whereas current smokers had a significantly higher BAaccel by 1.5387 years (β=1.5387; 95% CI: 1.1197–1.9577, p<0.001) (Table 3). The diagnosis of multicollinearity shows that the GVIF^1/2df^ values of each covariate in the fully adjusted model (Model 3) range from 1.01 to 1.31 (smoking status: 1.06; daily smoking amount: 1.08), all of which are below the threshold of 2, indicating that there is no serious multicollinearity problem in the model. Restricted cubic spline analysis showed no evidence of a nonlinear association between CPD and BAaccel (p for nonlinearity=0.285).

**Table 3:** Multivariable linear regression analyses of the associations of cigarettes per day and smoking status with GOLD biological age acceleration, NHANES 1999–2023 (N=6345–12941)

*Exposure*	*Model*	*β*	*95% CI*	*SE*	*p*	*Sample size* *(n)*
**Cigarettes per day** (continuous)	Model 1	0.0185	-0.0024–0.0394	0.0107	0.082	9761
	Model 2	0.069	0.0466–0.0915	0.0115	<0.001	7209
	Model 3	0.0763	0.0522–0.1003	0.0123	<0.001	6345
	Model 3 (MICE multiple imputation)	0.076	0.0486–0.1033	0.014	<0.001	9761
**Smoking status** (ref: never smoking)	Model 1 – former smoking	-0.7554	-1.0504 – -0.4604	0.1505	<0.001	12941
	Model 1 – current smoking	0.4228	-0.0637–0.9094	0.2482	0.089	12941
	Model 2 – former smoking	-0.1043	-0.3430–0.1345	0.1218	0.392	9618
	Model 2 – current smoking	1.3396	0.9284–1.7508	0.2098	<0.001	9618
	Model 3 – former smoking	-0.0546	-0.3381–0.2288	0.1446	0.706	8429
	Model 3 – current smoking	1.5387	1.1197–1.9577	0.2138	<0.001	8429

Biological age acceleration (BAaccel) was defined as the residual from a linear regression of GOLD BioAge on chronological age. Cigarettes per day was entered as a continuous variable, and never smoking was used as the reference category for smoking status. Model 1 included the exposure variable only, without covariate adjustment. Model 2 additionally adjusted for sex, race/ethnicity, education level, family poverty income ratio, body mass index, and marital status. Model 3 further adjusted for survey cycle, alcohol consumption, diabetes, hypertension, hyperlipidemia, and physical activity. Sample sizes decreased across models as additional covariates were included; participants with missing data for any covariate were excluded. Inference was based on ordinary least squares (OLS) regression with cluster-robust standard errors (SEs), clustered by primary sampling unit (PSU). A survey-weighted generalized linear model (svyglm) accounting for the complex survey design was used as a sensitivity analysis for variance estimation. The 95% CIs were calculated using t-distribution critical values (coeftest/confint). MICE: multiple imputation by chained equations. CI: confidence interval. A two-sided p<0.05 was considered statistically significant.

### Sensitivity analyses

**Figure 2:**
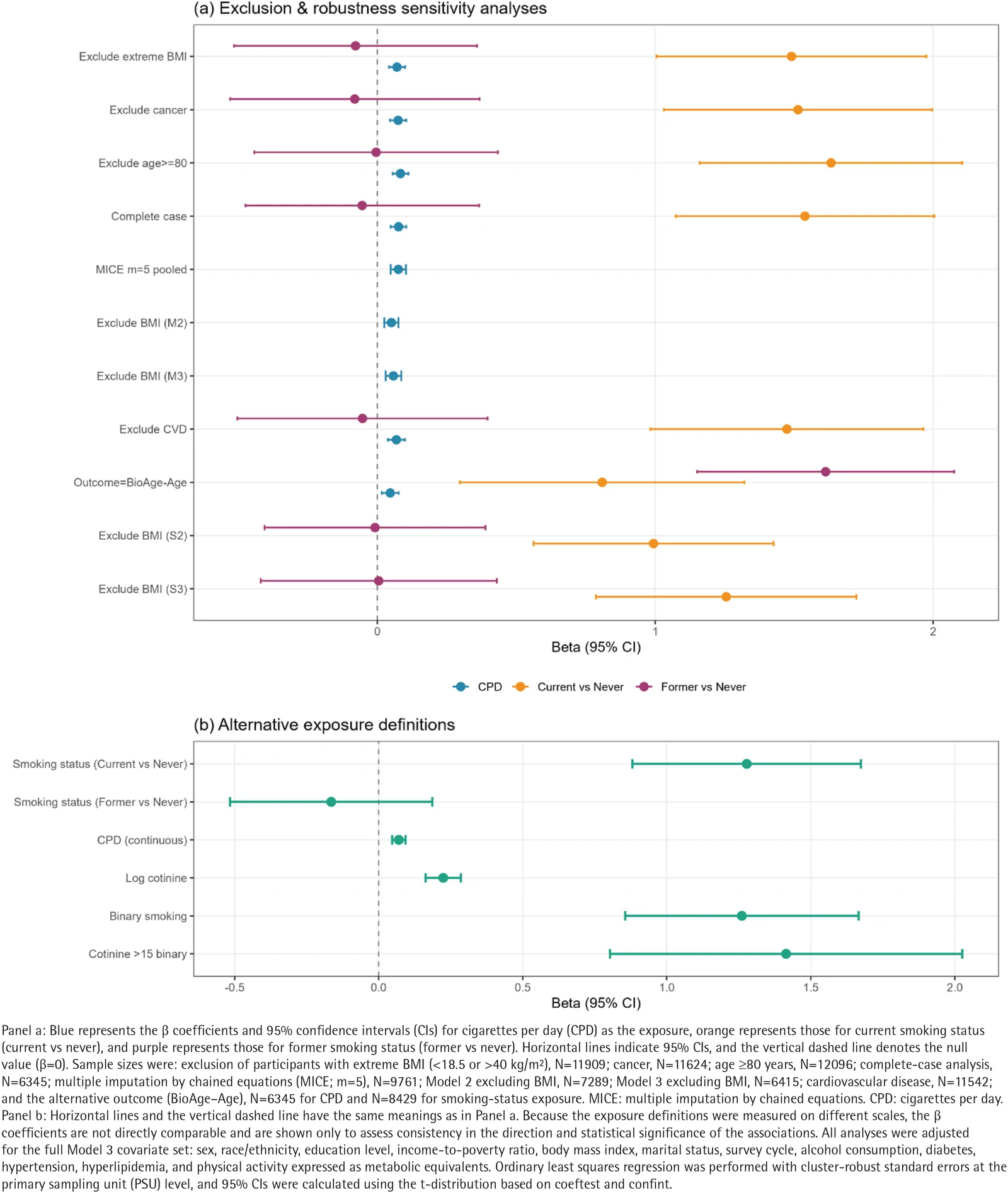
Forest plots of sensitivity analyses in NHANES 1999–2023: a) Exclusion and robustness sensitivity analyses; b) Sensitivity analyses using alternative exposure definitions. These included three-category smoking status (N=11303), continuous CPD (N=8429), log-transformed serum cotinine (N=5213), binary smoking status (N=8443), and a serum cotinine threshold of >15 ng/mL (N=4028)

To assess the robustness of the main findings, a series of sensitivity analyses were performed. After sequentially excluding participants with extreme BMI (<18.5 or >40 kg/m^2^), cancer, aged ≥80 years, or cardiovascular disease, the positive association between CPD and BAaccel remained significant, with β estimates ranging from 0.0686 to 0.0833 (all p<0.001). The corresponding associations for current smokers were also stable (β: 1.474–1.633, all p<0.001), whereas former smokers consistently showed no significant differences (all p>0.05). Complete case analysis and MICE imputation (m=5) yielded results consistent with the primary analysis. After removing BMI from the model, the CPD effect was slightly attenuated (β=0.0576; 95% CI: 0.030–0.086, p<0.001), and the current smoker effect also declined somewhat (β=1.2556; 95% CI: 0.787–1.724, p<0.001), while former smokers remained non-significant (p=0.982). However, when the outcome was replaced with the unstandardized biological age difference (BioAge–Age), the CPD effect was 0.0465 (β=0.0465; 95% CI: 0.016–0.077, p=0.003); the effect for current smokers weakened to 0.8092 years (β=0.8092; 95% CI: 0.297–1.321, p=0.002), and that for former smokers was 1.6133 years (β=1.6133; 95% CI: 1.151–2.076, p<0.001). This latter finding contrasts with the null result for former smokers in the main analysis (Figure 2a).

Results were also consistent when alternative smoking measures were used. In models using 3-category smoking status, current smokers had a 1.2778-year higher BAaccel than never smokers (β=1.2778; 95% CI, 0.881–1.674; p<0.001), whereas former smokers did not differ significantly from never smokers (p=0.357). The estimate for CPD was β=0.0699 (95% CI: 0.047–0.093; p<0.001). Log-transformed serum cotinine was positively associated with BAaccel (β=0.2241; 95% CI: 0.163–0.285; p<0.001). Binary comparisons of current versus never smoking and cotinine concentrations >15 ng/mL yielded results in the same direction as the primary analysis (β=1.2608; 95% CI: 0.856–1.666; p<0.001 and β=1.4144; 95% CI: 0.803–2.026; p<0.001, respectively) (Figure 2b).

At the statistical-method level, the standard error of the CPD coefficient was 0.0123 when estimated using ordinary least squares regression with PSU-clustered robust standard errors and 0.0179 when estimated using survey-weighted methods. E-value analysis indicated that an unmeasured confounder would need to have associations >1.5 with both the exposure and outcome to fully explain the observed association.

### Mediation analysis

For daily smoking amount as the independent variable, in the UA mediation model, the total effect (TE) was 0.0764 (95% CI: 0.0476–0.1049, p<0.001). The indirect effect via UA (ACME) was 0.0039 (95% CI: 0.0016–0.0066, p<0.001), the direct effect (ADE) was 0.0725 (95% CI: 0.0439–0.0998, p<0.001), and the mediating proportion was 5.1%. In the CRP mediation model, TE was 0.0785 (95% CI: 0.0346–0.1193, p=0.002); the indirect effect via CRP (ACME) was 0.0117 (95% CI: 0.0046–0.0210, p<0.001), ADE was 0.0668 (95% CI: 0.0238–0.1061, p=0.002), and the mediating proportion was 14.88%.

For current smoking status (with never smokers as reference), in the UA model, TE was 1.547 (95% CI: 1.0723–2.0235, p<0.001); the indirect effect via UA was 0.0371 (95% CI: 0.0020–0.0761, p=0.038), ADE was 1.51 (95% CI: 1.0383–1.9951, p<0.001), and the mediating proportion was 2.4%. In the CRP model, TE was 2.1157 (95% CI: 1.3378–2.9046, p<0.001); the indirect effect via CRP was 0.1945 (95% CI: 0.0898–0.3177 , p<0.001), ADE was 1.9212 (95% CI: 1.1311–2.7487, p<0.001), and the mediating proportion was 9.19 % (Table 4).

**Table 4:** Mediation effects of UA and CRP on the associations of different smoking patterns with GOLD biological age acceleration, NHANES 1999–2023 (N=2269–6353)

*Smoking* *pattern*	*Mediator*	*Pathway*	*ACME*	*ACME 95% CI*	*ACME* *p*	*ADE*	*ADE 95% CI*	*ADE p*	*TE*	*TE* *95% CI*	*TE* *p*	*Percent* *mediated*	*n*
**Cigarettes per day**	Uric acid (UA)	Oxidative stress	0.0039	0.0016–0.0066	<0.001	0.0725	0.0439–0.0998	<0.001	0.0764	0.0476–0.1049	<0.001	5.1	6343
	C-reactive protein (CRP)	Systemic inflammation	0.0117	0.0046–0.021	<0.001	0.0668	0.0238–0.1061	0.002	0.0785	0.0346–0.1193	0.002	14.88	2269
**Current smoking status**	Uric acid (UA)	Oxidative stress	0.0371	0.002–0.0761	0.038	1.51	1.0383–1.9951	<0.001	1.547	1.0723–2.0235	<0.001	2.4	6353
	C-reactive protein (CRP)	Systemic inflammation	0.1945	0.0898–0.3177	<0.001	1.9212	1.1311–2.7487	<0.001	2.1157	1.3378–2.9046	<0.001	9.19	2272

Mediation analyses were conducted using the R mediation package with 1000 bootstrap simulations. Biological age acceleration (BAaccel) was defined as the residual from a linear regression of GOLD BioAge on chronological age. Cigarettes per day was entered as a continuous variable, and never smoking was used as the reference category for smoking status. ACME: average causal mediation effect (indirect effect). ADE: average direct effect. TE: total effect, calculated as ACME + ADE; proportion mediated, ACME/TE. Mediation models were adjusted for all Model 3 covariates, including sex, race/ethnicity, education level, income, body mass index, marital status, survey cycle, alcohol consumption, diabetes, hypertension, hyperlipidemia, and physical activity. Mediation analyses were based on complete-case subsamples (n values are shown in the table). Because the current mediation package does not support complex survey weights, all 95% CIs were derived from bootstrap resampling. A two-sided p<0.05 was considered statistically significant. CI: confidence interval.

### Subgroup analyses

**Figure 3:**
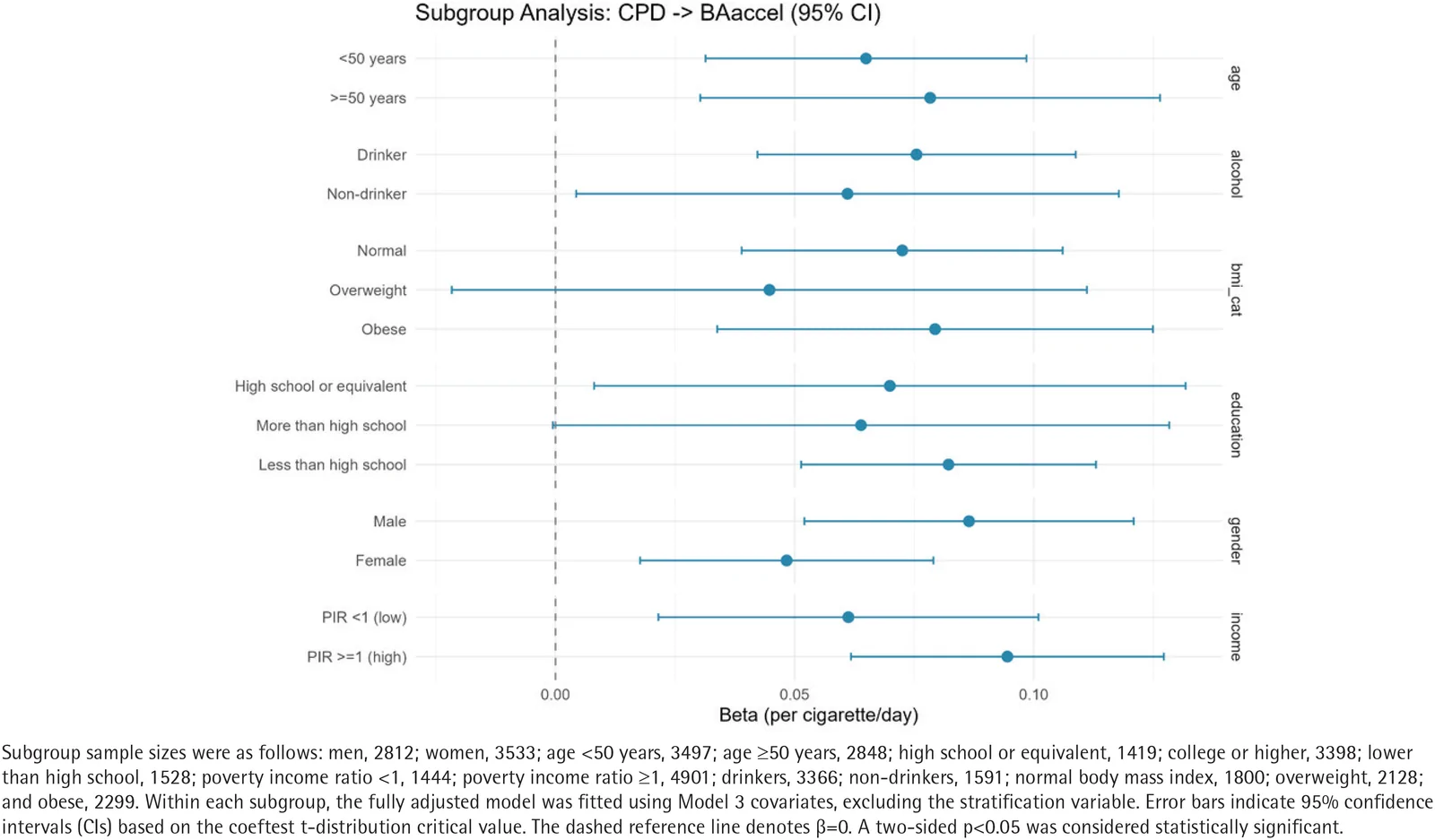
Forest plot of subgroup analyses for the association between daily cigarette consumption and GOLD BioAge acceleration, NHANES 1999–2023

Associations between CPD and GOLD BioAge acceleration did not differ significantly by sex, age, education level, income, alcohol use, or BMI category (all p for interaction >0.05) (Figure 3).

**Figure 4:**
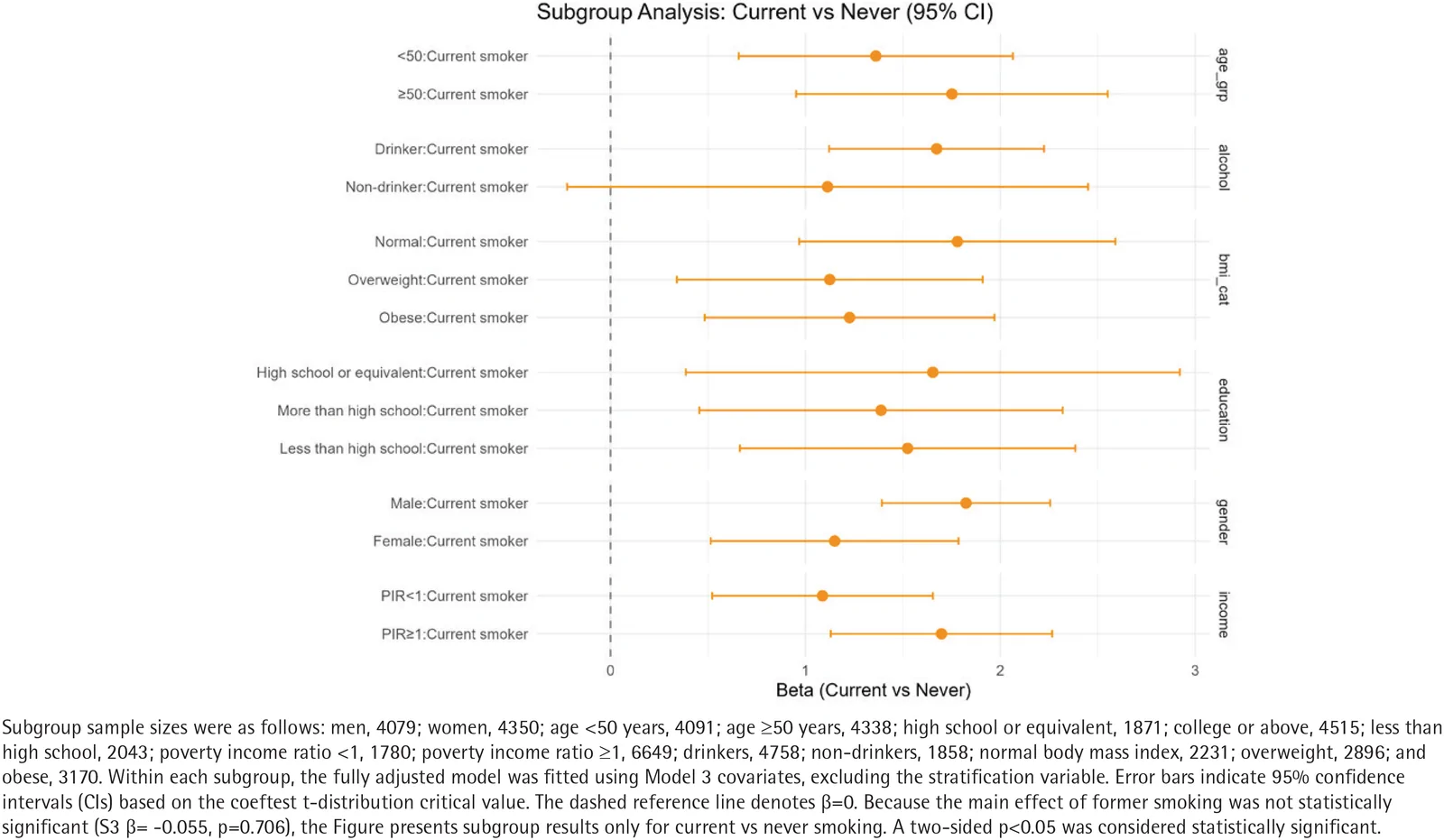
Forest plot of subgroup analyses for the association between current smoking status and GOLD BioAge acceleration, NHANES 1999–2023

With never smokers as the reference group, the association between current smoking and GOLD BioAge acceleration differed significantly by age (p for interaction=0.019). The association was stronger among participants aged ≥50 years (β=1.7519). No significant interactions were observed by sex, education level, income, alcohol use, or BMI category (all p for interaction >0.05). Because former smoking was not significantly associated with BAaccel in the primary analysis (p=0.706), subgroup analyses of smoking status were limited to current versus never smoking (Figure 4).

## DISCUSSION

In this nationally representative study of 12941 US adults from 12 NHANES cycles conducted between 1999 and 2023, greater smoking intensity and current smoking were associated with higher GOLD BioAge acceleration. These findings extend prior research on smoking-related biological aging by applying a recently developed biomarker-based aging measure that is readily calculated from routine clinical data.

Smoking behavior is positively associated with GOLD biological age acceleration. Daily smoking volume also exhibits a positive association with this acceleration, supporting a cumulative effect of tobacco exposure on aging. In categorical analyses, current smokers showed significantly greater GOLD biological age acceleration than never smokers. These complementary findings from continuous and categorical exposure measures corroborate the robust association between smoking behavior and GOLD biological age acceleration. The consistency of these findings when serum cotinine was used as an objective marker, supports the validity of the self-reported smoking measures. Our results are consistent with those of Perez-Garcia et al.^23^, who used 12 DNAm age biomarkers and found dose-dependent associations between smoking and epigenetic age acceleration among current and former smokers. Zhong et al.^24^ also used serum cotinine as an objective measure of tobacco exposure and identified a dose–response association with PhenoAge acceleration, providing indirect support for our findings. Similarly, an analysis of NHANES 1999–2002 data reported dose–response associations between cotinine and several epigenetic aging measures^25^.

Former smokers did not differ significantly from never smokers in BAaccel, whereas current smokers had significantly higher BAaccel. This pattern suggests that smoking cessation may be associated with attenuation of smoking-related biological age acceleration, although the cross-sectional design does not establish reversibility. This interpretation is consistent with the NHANES analysis by Perez-Garcia et al.^23^, which found that epigenetic age acceleration decreased with longer smoking-cessation duration. Other studies using multiple epigenetic clocks reported cessation-related differences of 10.17 years for GrimAge2 acceleration and 2.64 years for PhenoAge acceleration^21^. Reversible DNAm changes at the AHRR locus after smoking cessation provide additional biological plausibility^26-28^.

The associations remained broadly consistent across multiple sensitivity analyses, including alternative exposure definitions, analytic methods, exclusion criteria, and outcome specifications. This consistency strengthens the epidemiological evidence linking smoking with accelerated biological aging and supports the potential relevance of smoking cessation to healthy aging.

CRP accounted for a larger mediated proportion than UA in the present analyses, suggesting that systemic inflammation may contribute more strongly than oxidative stress to the observed association between smoking and biological age acceleration. Mechanistic evidence provides biological plausibility for this interpretation. Shreya et al.^29^ reported that cigarette smoke extract downregulated the telomere-stability genes TRF2 and POT1 and upregulated the inflammatory protein IFN-γ, linking cigarette smoke exposure with telomere dysfunction and inflammatory responses. Other studies have reported elevated inflammatory and oxidative stress markers among smokers^30^, and additional evidence supports potential roles for both processes in smoking-related aging^31,32^. Nevertheless, because the mediation analyses were based on cross-sectional data, temporal ordering and causal mediation cannot be established. These potential mechanisms should therefore be examined in longitudinal cohorts and experimental studies.

The association between CPD and BAaccel was consistent across the examined subgroups. In contrast, the association between current smoking and BAaccel differed by age and was stronger among adults aged ≥50 years than among those aged <50 years. This pattern may reflect cumulative smoking exposure^33^ and age-related reductions in DNA repair capacity together with chronic low-grade inflammation, which could increase susceptibility to smoking-related damage^29,34,35^. However, Cui et al.^36^ reported the opposite pattern in the UK Biobank, where biological age acceleration was more pronounced among younger smokers and absent in the oldest group. Survivor bias, differences in biological age algorithms, and population characteristics may help explain these discrepant findings. The stronger association observed in older adults should therefore be interpreted cautiously and examined further in longitudinal studies.

### Strengths and limitations

This study has several strengths. To our knowledge, it is the first to apply the GOLD BioAge algorithm to examine the association between smoking behaviors and biological aging, thereby extending the evidence supporting its application in population-based research. The analysis included nationally representative data from 12 NHANES survey cycles spanning 1999–2023, which enhanced the population coverage and generalizability of the findings. Smoking exposure was characterized using both smoking status and the number of cigarettes smoked per day, allowing different dimensions of smoking behavior to be evaluated. The consistency of the findings was further examined through a series of sensitivity analyses. In addition, complementary statistical approaches were employed, including cluster-robust standard errors and survey-weighted generalized linear models, to account for the complex sampling design of NHANES and assess the robustness of the statistical inference. This study has several limitations. First, smoking exposure was based on self-report, which may introduce misclassification bias. Although serum cotinine validation (>15 ng/mL) showed high concordance with self-reported status, reporting bias cannot be completely ruled out. Second, NHANES is cross-sectional, with exposure and outcome measured at a single time point, making it difficult to establish causal temporality and precluding assessment of dynamic changes in biological aging. Consequently, the causal inference from the mediation analysis is also limited, and the proposed pathway hypotheses require further support from experimental or longitudinal cohort studies. Third, despite adjustment for multiple confounders, the potential for residual confounding from unmeasured factors, such as psychosocial factors, sleep, and dietary habits, cannot be discounted. Finally, the NHANES sample represents non-institutionalized US civilian adults, which limits generalizability of our findings. Moreover, the GOLD BioAge algorithm was derived from an external independent sample, and its applicability across different racial and socioeconomic groups needs further validation.

## CONCLUSIONS

Based on cross-sectional NHANES data, this study found that smoking behavior was positively associated with biological age acceleration. This association was consistent for both daily smoking amount and current smoking status. Prospective longitudinal studies are needed to determine whether smoking cessation is associated with improvements in biological age acceleration.

## Data Availability

The data supporting this research are available from the authors on reasonable request.
